# Association between the composite dietary antioxidant index and non-alcoholic fatty liver disease: evidence from National Health and Nutrition Examination Survey 2005–2016

**DOI:** 10.3389/fnut.2025.1473487

**Published:** 2025-01-23

**Authors:** Yidian Fu, Chao Jiang, Zonglin Li, Xiangyun Shi, Peiyuan Lv, Jingbo Zhang

**Affiliations:** ^1^Graduate School of Hebei Medical University, Shijiazhuang, Hebei, China; ^2^Department of Neurology, Hebei General Hospital, Shijiazhuang, Hebei, China; ^3^Department of Psychosomatic Medicine, Hebei General Hospital, Shijiazhuang, Hebei, China; ^4^Department of Medical Laboratory, Liaocheng Hospital of Traditional Chinese Medicine, Liaocheng, Shandong, China; ^5^College of Geography and Resources, Sichuan Normal University, Chengdu, China; ^6^Department of Dermatology, The First Affiliated Hospital of Chongqing Medical University, Chongqing, China

**Keywords:** composite dietary antioxidant index, non-alcoholic fatty liver disease, NHANES, US fatty liver index, hepatic steatosis index

## Abstract

**Importance:**

Oxidative stress contributes to the progression of non-alcoholic fatty liver disease (NAFLD). Antioxidants from food can reduce NAFLD incidence, and the Composite Dietary Antioxidant Index (CDAI) measures total antioxidant capacity (TAC). However, the relationship between CDAI and NAFLD in the US adult population remains unclear.

**Objective:**

To assess whether CDAI is associated with NAFLD in US adults.

**Design, setting, and participants:**

This population-based cross-sectional study used data on US adults from the National Health and Nutrition Examination Survey (NHANES) 2005–2016 cycles. Data were analyzed from January to February 2024.

**Exposures:**

CDAI obtained from the dietary intake questionnaire.

**Main outcomes and measures:**

The main outcome was NAFLD which defined by the US fatty liver score (USFLI) ≥30. Sampling weights were calculated according to NHANES guidelines.

**Results:**

Among 9,746 adults included in this study [mean age, 48.3 years; 4,662 (47.6%) males], 3,324 (33.0%) were classified as having NAFLD using USFLI. In the fully adjusted of multivariable logistic regression, CDAI was negatively associated with NAFLD (odds ratio [OR], 0.95; 95% CI, 0.93–0.98). Furthermore, individuals in the highest quartile of CDAI were 34% less likely to have NAFLD compared to those in the lowest quartile (OR, 0.66; 95% CI, 0.52–0.85). In subgroup analyses, CDAI was inversely associated with NAFLD among participants with a BMI <25 (OR, 0.89; 95% CI, 0.83–0.95) and without metabolic syndrome (OR, 0.93; 95% CI, 0.91–0.96). The interaction tests revealed significant differences in these subgroups (*P* for interaction = 0.04 for BMI and 0.003 for metabolic syndrome). Sensitivity analyses confirmed this association using the hepatic steatosis index (HSI) to define NAFLD, applying unweighted logistic regression, adjusting for physical activity or after excluding non-Hispanic Black participants, and after excluding medications known for their potential hepatotoxic effects.

**Conclusions and relevance:**

In this cross-sectional study based on six cycles (2005–2016) of the NHANES, CDAI was negatively associated with NAFLD in US adult population. This association highlights the potential for dietary interventions to reduce NAFLD incidence and underscores the need for future research, including clinical trials and mechanistic studies, to further explore the role of dietary antioxidants in NAFLD prevention and management.

## Introduction

Non-alcoholic fatty liver disease (NAFLD) encompasses a broad spectrum of liver pathologies, spanning from simple steatosis (non-alcoholic fatty liver, NAFL) and non-alcoholic steatohepatitis (NASH) to fibrosis and cirrhosis (1). With the prevalence of obesity, type 2 diabetes mellitus (T2DM), and metabolic syndrome, NAFLD has emerged as the predominant chronic liver ailment globally (2). NAFLD affects ~25% of the world's population (3). In 2016, while China had the greatest number of NAFLD cases (243.67 million), its prevalence was lower (17.6%) compared to other countries, with the highest prevalence found in the US at 26.3%, affecting 85.3 million individuals (4). In the United States, it is anticipated that NASH will become the foremost indication for liver transplantation in the foreseeable future (2, 5). A previous study of NAFLD model estimated that ~800,000 liver-related deaths will occur in the NAFLD population from 2015 to 2030 (5). Given the absence of universally accepted pharmaceutical or surgical interventions for NAFLD, lifestyle modifications, including dietary adjustments, are commonly advocated for both the prevention and management of NAFLD (6). However, challenges such as poor adherence, difficulty in sustaining long-term changes, and the need for individualized approaches often limit their effectiveness, underscoring the importance of identifying specific dietary components, such as antioxidants, that may offer targeted and practical interventions.

NAFLD is a complex and multifactorial disorder linked to a plethora of genetic, epigenetic, and environmental determinants. Despite ongoing research efforts, the intricacies of its pathogenesis remain incompletely understood (7). Presently, the “multiple-hit” hypothesis stands as a prominent theoretical framework for elucidating NAFLD pathogenesis, positing that several factors may concurrently contribute to disease development. Among the various factors that contribute to the “multiple hits” is oxidative stress, which is considered the main contributor to liver injury and disease progression in NAFLD (8, 9). Oxidative stress is a key factor in the progression of NASH, as it increases lipid peroxidation in cell membranes and activates stellate cells in the liver, leading to fibrosis, chronic inflammation, and apoptosis. Reactive oxygen species (ROS) and lipid peroxidation directly damage hepatocytes by affecting membranes, proteins, and DNA (10). Inflammation is closely linked to NAFLD (11), as the accumulation of liver fat is associated with the production of inflammatory markers. Studies have demonstrated that key inflammatory markers, including C-reactive protein (CRP) and interleukins (ILs), are significantly elevated in individuals diagnosed with NAFLD and NASH (12). Dietary antioxidants have been shown to be vital in regulating lipid homeostasis, as well as the expression and activity of metabolism-related proteins, which influence lipid synthesis, oxidation, peroxidation, and inflammation (13). When antioxidant and anti-inflammatory defenses are depleted, a chronic state of steatohepatitis develops. Hence, the intake of antioxidant-rich diet may reduce risk, severity, or progression of this disease. An expanding body of research indicates that elevated levels of dietary antioxidants intake are associated with a decreased risk for NAFLD or for disease progression (14–18). Qi et al. found that increasing dietary intake of vitamin E is beneficial in preventing NAFLD, especially among individuals without hyperlipidemia (19). Oliveira et al. reported that a higher dietary total antioxidant capacity (TAC) was observed in patients with lower hepatic injury (ballooning) (10), suggesting that diets naturally rich in antioxidants may play a role in reducing free radical production and, consequently, oxidative stress. Moreover, higher intake levels of carotenoids were associated with lower odds of NAFLD and could help reverse hepatic steatosis (13, 16).

The Composite Dietary Antioxidant Index (CDAI) is a composite score employed to assess an individual's dietary total antioxidant capacity (TAC). The CDAI is an individual antioxidant index based on a combination of dietary antioxidant, including vitamin A, vitamin C, vitamin E, zinc, selenium, and carotenoids (20). Previous studies have indicated that a high CDAI is correlated with a decreased risk of several types of cancer, diabetes, as well as all-cause and cardiovascular mortality (21–24). The relationship between CDAI and dietary antioxidants may vary across populations due to factors like BMI, sex, and health conditions. For example, higher BMI may enhance the benefits of dietary antioxidants in stroke prevention (25), and CDAI has been shown to protect against constipation in males but not females (26). However, the relationship between CDAI and NAFLD remains unclear in the general population of U.S. adults.

We hypothesize that higher CDAI is associated with lower odds of NAFLD, as assessed using the US fatty liver index (USFLI). Therefore, the objective of this study was to investigate this relationship among a nationally representative sample of US adults in the National Health and Nutrition Examination Survey (NHANES).

## Materials and methods

### Study design

This study utilized publicly available data obtained from the National Health and Nutrition Examination Survey (NHANES), a comprehensive cross-sectional survey administered by physicians and highly trained medical personnel. The survey encompasses questionnaires, physical examinations, and laboratory data. NHANES aims to ascertain the prevalence and identify risk factors associated with major diseases in the U.S. population. Released biannually, the survey provides data collected from participants across the United States, selected through a sophisticated multistage, stratified sampling method. This study followed the Strengthening the Reporting of Observational Studies in Epidemiology (STROBE) reporting guidelines for cross-sectional studies.

### Study population

**Figure 1:**
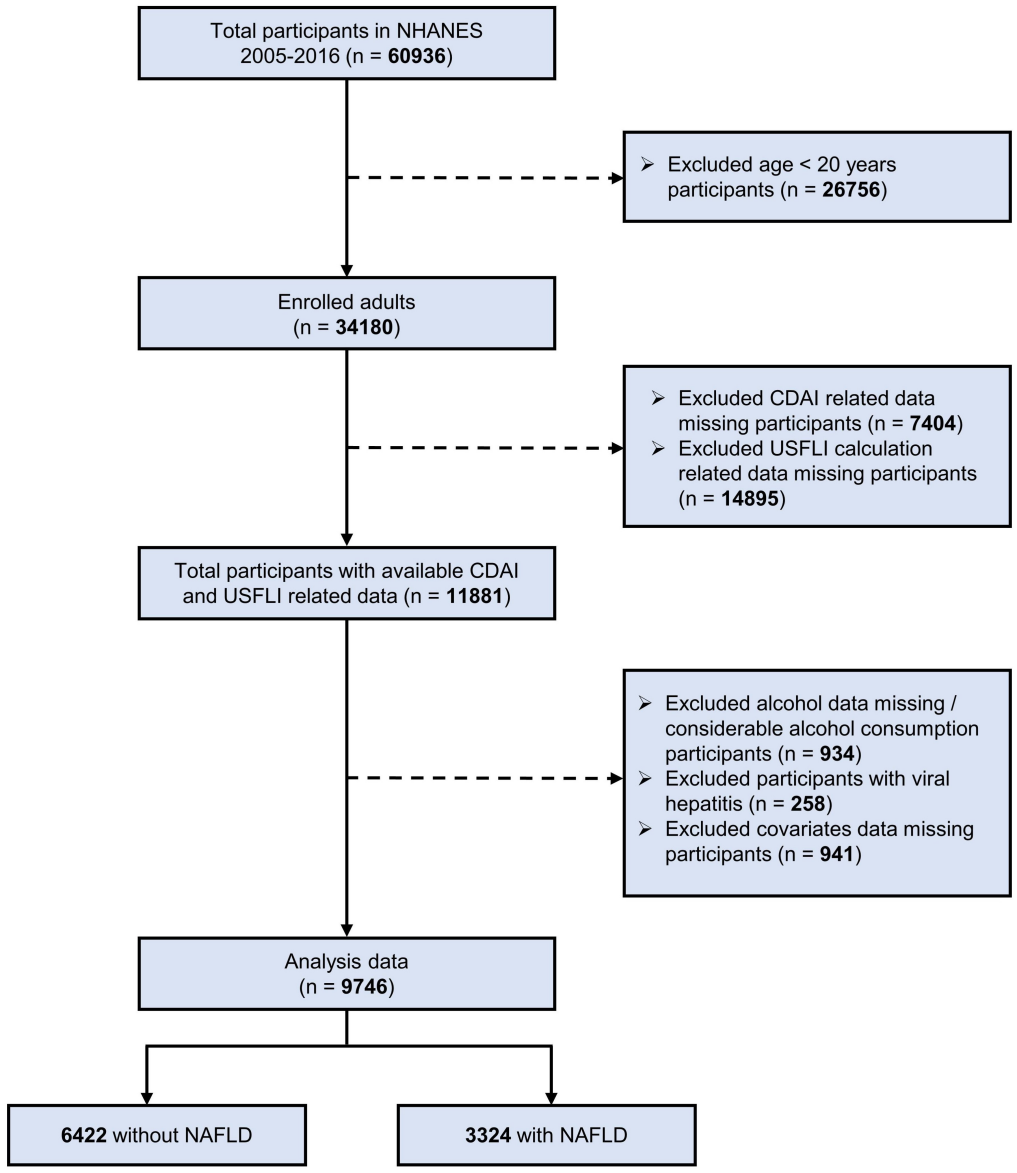
Flow chart of the screening and enrollment of study participants. NHANES, National Health and Nutrition Examination Survey; NAFLD, non-alcoholic fatty liver disease; CDAI, composite dietary antioxidant index; USFLI, US fatty liver index.

In present study, we utilized data from six NHANES cycles (2005–2006, 2007–2008, 2009–2010, 2011–2012, 2013–2014, and 2015–2016) that involved a total of 60,936 participants. We excluded participants younger than 20 years, leaving 34,180 adults. Participants were excluded by the following criteria (18, 27–29): missing data for the CDAI calculation; missing data for the calculation of US fatty liver index (USFLI) score; missing data for alcohol consumption or presence of considerable alcohol consumption (>21 drinks per week for male and >14 drinks per week for female); participants with the hepatitis B surface antigen or hepatitis C antibodies; missing data for covariates. Therefore, a total of 9,746 participants was included in the present study, and the flowchart of enrollment is presented in Figure 1. Referring to previous NHANES literature (30, 31), data from the NHANES project was analyzed using a complex stratified sampling method.

### Definition of primary exposure

Diet affects the CDAI. CDAI data were derived from the two 24-h dietary recall survey of NHANES participants. The first 24h was recorded face-to-face at a mobile examination center, and the second 24 h was recorded by telephone 3–10 days later. The University of Texas Food Intake Analysis System and the United States Department of Agriculture Survey Nutrient Database were used to assess the dietary nutrient intake (32). The nutritional estimates did not include any nutrients obtained from dietary supplements or medications. The average of the two 24-h intakes was taken as the daily dietary intake for the present study (33).

The CDAI was calculated from the mean dietary intake of vitamin A, vitamin C, vitamin E, zinc, selenium, and carotenoids obtained from two 24-h recalls using a modified version developed by Wright et al. (20). Six antioxidants were standardized by subtracting the mean and dividing by the standard deviation. Then the CDAI was calculated based on the sum of these standardized values.


$$\begin{array}{l} CDAI=\;\overset{6}{\underset{i=1}{\sum }}\frac{X_{i}-\mu_{i}}{s_{i}}\; \end{array}$$

In this formula, *X*_*i*_ represents the individual daily intake of antioxidant components; μ_*i*_ represents the mean of *X*_*i*_; *s*_*i*_ represents the standard deviation for μ_*i*_ (33–35).

### Definition of outcome

NAFLD was defined according to the USFLI which was developed using the NHANES database, which was moderately improved accuracy compared to the Fatty Liver Index (FLI) in the multiethnic US population (18). And it has been validated and used in several previous studies (36–38). USFLI was developed based on race/ethnicity, age, gamma-glutamyl transferase (GGT), waist circumference (WC), fasting insulin, and fasting glucose with the following formula:


$$\begin{array}{l} USFLI=\;\frac{e^{\begin{array}{c} (-0.8073\times non-Hispanic\;Black+0.3458\times Mexican\;American+0.0093\times Age\\ +0.6151\times \ln GGT+0.0249\times WC+1.1792\times \ln\;insulin\\ +0.8242\times \ln glucose-14.7812) \end{array}}}{1+\;e^{\begin{array}{c} (-0.8073\times non-Hispanic\;Black+0.3458\times Mexican\;American+0.0093\times Age\\ +0.6151\times \ln GGT+0.0249\times WC+1.1792\times \ln insulin\\ +0.8242\times \ln glucose-14.7812) \end{array}}}\\ \times 100 \end{array}$$

Scores range from 0 to 100. In this study, a USFLI score ≥ 30 was considered to have NAFLD as suggested by Ruhl and Everhart (18), with an area under the receiver operating characteristic curve (AUROC) of 0.8 (sensitivity: 62%; specificity: 88%).

### Covariates

Based on the literature, the following covariates were selected, including: age, sex, race/ethnicity, education level, marital status, family income, body mass index (BMI), alcohol drinking status, smoking status, diabetes, hypertension, metabolic syndrome, and total energy intake (39, 40). As used by NHANES, we divided race/ethnicity into Mexican American, other Hispanic, non-Hispanic White, non-Hispanic Black and Other (including multiracial). Education level was divided into two groups (high school or below and greater than high school). The marital status was classified as married, never married, living with a partner, and others (including divorced, widowed, and separated). Family income was categorized into 3 levels (< 1.3, 1.3–3.5, and ≥3.5) based on the family poverty income ratio (PIR). BMI was divided into 3 levels (< 25, 25–30, and ≥30 kg/m^2^). Alcohol drinking status was determined by the following survey question, “In any 1 year, have you had at least 12 drinks of any type of alcoholic beverage?” Participants who answered “yes” were defined as alcohol drinkers. Serum cotinine concentration was utilized as a proxy for environmental tobacco exposure and categorized into active/secondhand smoker (>0.011 ng/mL) and non-smoker (≤ 0.011 ng/mL). Diabetes was defined as using antidiabetic medication or a fasting glucose level equal or >126 mg/dL. Hypertension was defined as using antihypertensive medication or average systolic blood pressure ≥140 mmHg and/or average diastolic blood pressure ≥80 mmHg. Metabolic syndrome was defined based on the Adult Treatment Panel III criteria in 2005 as having at least 3 of the following: waist circumference >102 cm in men or 88 cm in women, triglyceride level >150 mg/dL, high-density lipoprotein cholesterol < 40 mg/dL in men or < 50 mg/dL in women, systolic blood pressure at least 130 mm Hg or diastolic blood pressure at least 85 mm Hg or taking hypertension medications, or fasting plasma glucose level at least 100 mg/dL or taking diabetes medications (41, 42). Total energy intake was calculated by averaging energy intake collected during two 24-h total nutrient recall interviews

### Statistical analysis

According to NHANES analytic guidelines, complex sampling design and sampling weights were considered in our analyses (43). The characteristics of participants are described as means (95% CIs) for continuous variables and percentage frequencies (95% CIs) for categorical variables. Continuous data were compared using *t*-tests, and categorical data were compared by the χ^2^ test. These means and frequencies can be generalized to the US adult population. No imputation method was used due to the percentage of missing data was small for any variable.

Odds ratios (ORs) and 95% CIs were calculated to assess the association between CDAI/antioxidant components and NAFLD using weighted logistic regression models. Given that the values of antioxidant components intake were skewed, a logarithmic change was performed before statistical analysis to ensure a normal distribution. And the CDAI was generally converted into categorical variables according to quartiles, and the *P*-values for the trend were calculated. Three models were used in this study. Model 1 was the crude model with no covariates adjusted. Model 2 was adjusted for age, sex, and race/ethnicity. Model 3 was the fully adjusted model which including age, sex, race/ethnicity, education level, marital status, PIR, BMI, alcohol drinking status, serum cotinine, diabetes, hypertension, metabolic syndrome, and total energy intake.

In addition, interaction and subgroup analyses of association between CDAI and NAFLD were also performed according to sex (male, female), age (20–40, 40–60, ≥60 years), BMI (< 25, 25–30, and ≥30 kg/m^2^), diabetes (no, yes), hypertension (no, yes), and metabolic syndrome (no, yes) using logistic regression models.

To ensure the robustness of our research findings, we adopted the methods used by Ruan et al. (36) and conducted several sensitivity analyses. Initially, to assess the potential hepatotoxicity of certain pharmacological agents, we conducted a sensitivity analysis excluding participants who had been administered methotrexate, acitretin, pioglitazone, liraglutide, semaglutide, atorvastatin, or aspirin (44–53). Subsequent to this, drawing upon extant literature which posited diminished prevalence rates of NAFLD and suboptimal diagnostic precision of USFLI with the non-Hispanic Black cohort (18, 36, 54, 55), we implemented a sensitivity analysis that omitted individuals belonging to this demographic. Finally, in an endeavor to mitigate the possibility of misclassification stemming from USFLI scores, we performed an additional sensitivity analysis utilizing the hepatic steatosis index (HSI) (56). Here, we defined the NAFLD as an HSI score ≥ 36 in present study with the following formula:


$$\begin{array}{r} HSI=8\times \frac{AST}{ALT}+BMI+2\left(if\;female\right)\\ +2\left(if\;diabetes\;mellitus\;present\right) \end{array}$$

Considering the calculation if HSI was based on BMI, BMI was not included as a covariate in this model to avoid the over adjustment. Ultimately, numerous previous studies have examined data from the NHANES to explore risk factors associated with various diseases, and some researchers have utilized weighted analysis methods, while others have employed unweighted approaches. Although NHANES employed complex sampling techniques to improve the representativeness and applicability of findings, conclusions derived from weighted and unweighted analyses can occasionally differ. Therefore, we conducted a sensitivity analysis using unweight data. We also further adjusted for physical activity which assessed by metabolic equivalent (MET) in a typical week to ensure the robustness of our results.

All statistical analysis was performed with R (version 4.1.3, R Project for Statistical Computing, Vienna, Austria) and EmpowerStats (version 4.1, Boston, Massachusetts). In all tests, *P* < 0.05 (2-sided) was considered to indicate statistical significance.

## Results

### Basic characteristics of the participants

A total of 9,746 participants (4,662 males [47.6%; 95% CI, 46.6–48.7%] and 5,082 females [52.4%; 95% CI, 51.3–53.4%]; 6422 without NAFLD [67.0%; 95% CI, 65.4–68.5%] and 3,324 with NAFLD [33.0%; 95% CI, 31.5–34.6%]; mean age: 48.3 years [95% CI, 47.7–48.9]) were included. Participants were excluded if they were under 20 years of age, had missing data for CDAI or USFLI calculations, reported excessive alcohol consumption, tested positive for hepatitis B or C, or had incomplete covariate information. The weighted baseline characteristics of included participants are shown in Table 1. Age, BMI, total energy intake, and zinc intake were lower in the participants without NAFLD vs. who with NAFLD (*P* < 0.05). Family PIR, education level, vitamin E intake, vitamin C intake, and Selenium intake were higher in the participants without NAFLD (*P* < 0.05). In the terms of alcohol drinking status, moderate-drinkers were more often in participants without NAFLD compared with their counterpart (*P* < 0.05). And participants with NAFLD were more likely to be Mexican American (*P* < 0.05). Compared with participants without NAFLD, those with NAFLD had a higher prevalence of diabetes (1,026 [26.6%; 95% CI, 24.5–28.7%] vs. 542 [5.7%; 95% CI, 5.0–6.5%]), hypertension (2,112 [62.2%; 95% CI, 59.8–64.5%] vs. 2,465 [33.5%; 95% CI, 31.7–35.2%]), and metabolic syndrome (1,846 [54.1%; 95% CI, 51.4–56.8%] vs. 802 [10.8%; 95% CI, 10.0–11.7%]). In addition, no significant differences were observed for serum cotinine, vitamin A intake, and carotenoids intake.

**Table 1:** Characteristic of participants in the NHANES 2005–2016 cycles.

**Characteristic**	**Participants** ^**a**^			
	**Total (*****N*** = **9,746)**	**Without NAFLD (*****N*** = **6,422)**	**With NAFLD**^b^ **(*****N*** = **3,324)**	***P-*** **value**
Age, mean (95% CI), y	48.3 (47.7, 48.9)	46.3 (45.6–47.0)	52.4 (51.6–53.2)	< 0.001
**Sex**				
Male	4,662 (47.6) [46.6–48.7]	2,805 (43.0) [41.6–44.4]	1,857 (57.1) [54.9–59.3]	< 0.001
Female	5,084 (52.4) [51.3–53.4]	3,617 (57.0) [55.6–58.4]	1,467 (42.9) [40.7–45.1]	
**Race/ethnicity** ^c^				
Mexican American	1,485 (7.4) [6.2–8.8]	712 (5.6) [4.7–6.6]	773 (11.1) [9.0–13.5]	< 0.001
Other Hispanic	887 (4.8) [3.8–5.9]	573 (4.9) [3.9–6.0]	314 (4.6) [3.5–5.9]	
**Non-Hispanic**				
White	4,726 (72.5) [69.6–75.1]	3,051 (71.6) [68.7–74.2]	1,675 (74.3) [70.9–77.4]	
Black	1,825 (9.3) [8.0–10.8]	1,455 (11.2) [9.2–13.0]	370 (5.5) [4.6–6.6]	
Other race/multiracial	823 (6.0) [5.4–6.8]	631 (6.8) [5.9–7.7]	192 (4.6) [3.9–5.4]	
**Education level**				
High school or below	4,413 (37.2) [34.9–39.5]	2,661 (34.1) [31.8–36.6]	1,752 (43.4) [40.8–46.1]	< 0.001
Great than high school	5,333 (62.8) [60.5–65.1]	3,761 (65.9) [63.4–68.2]	1,572 (56.6) [53.9–59.2]	
**Marital status**				
Married	5,328 (59.4) [57.6–61.2]	3,388 (58.2) [56.0–60.2]	1,940 (61.9) [59.4–64.3]	< 0.001
Never married	1,614 (15.9) [14.6–17.2]	1,236 (17.8) [16.3–19.4]	378 (11.9) [10.3–13.8]	
Living with partner	748 (7.5) [6.6–8.6]	522 (8.1) [7.0–9.3]	226 (6.4) [5.5–7.5]	
Others^d^	2,056 (17.2) [16.2–18.2]	1,276 (16.0) [15.0–17.0]	780 (19.8) [17.9–21.8]	
**Family PIR**				
< 1.3	2,841 (19.2) [17.5–20.6]	1,751 (18.2) [16.6–19.9]	1,090 (20.6) [18.8–22.6]	< 0.001
1.3–3.5	3,748 (36.6) [35.1–38.2]	2,463 (35.6) [33.9–37.4]	1,285 (38.7) [36.4–41.1]	
≥3.5	3,157 (44.4) [42.1–46.6]	2,208 (46.2) [43.8–48.6]	949 (40.7) [37.9–43.5]	
**BMI, kg/m** ^2^				
< 25	2,772 (29.2) [27.8–30.7]	2,643 (42.0) [40.3–43.7]	129 (3.3) [2.6–4.2]	< 0.001
25–30	3,275 (33.9) [32.8–34.9]	2,410 (38.6) [37.1–40.0]	865 (24.3) [22.6–26.1]	
≥30	3,699 (36.9) [35.5–38.4]	1,369 (19.5) [18.2–20.8]	2,330 (72.4) [70.5–74.2]	
**Alcohol drinking status** ^e^				
No	2,804 (23.8) [22.1–25.5]	1,804 (22.6) [21.0–24.4]	1,000 (26.1) [23.9–28.4]	< 0.001
Yes	6,942 (76.2) [74.5–77.9]	4,618 (77.4) [75.6–79.0]	2,324 (73.9) [71.6–76.1]	
**Serum cotinine** ^f^				
≤ 0.011 ng/mL	2,685 (29.5) [27.6–31.4]	1,774 (30.1) [28.0–32.3]	911 (28.1) [25.7–30.7]	0.13
>0.011 ng/mL	7,061 (70.5) [74.5–77.9]	4,648 (69.9) [67.7–72.0]	2,413 (71.9) [69.3–74.3]	
**Diabetes**				
No	8,178 (87.4) [86.4–88.3]	5,880 (94.3) [93.5–95.0]	2,298 (73.4) [71.3–75.5]	< 0.001
Yes	1,568 (12.6) [11.7–13.6]	542 (5.7) [5.0–6.5]	1,026 (26.6) [24.5–28.7]	
**Hypertension**				
No	5,169 (57.1) [55.5–58.7]	3,957 (66.5) [64.8–68.3]	1,212 (37.8) [35.5–40.2]	< 0.001
Yes	4,577 (42.9) [41.3–44.5]	2,465 (33.5) [31.7–35.2]	2,112 (62.2) [59.8–64.5]	
**Metabolic syndrome**				
No	7,098 (74.9) [73.6–76.1]	5,620 (89.2) [88.3–90.0]	1,478 (45.9) [43.2–48.6]	< 0.001
Yes	2,648 (25.1) [23.9–26.4]	802 (10.8) [10.0–11.7]	1,846 (54.1) [51.4–56.8]	
Total energy intakes mean (95% CI), kcal/day	2,081.9 (2,061.6–2,102.2)	2,055.8 (2,034.2–2,077.5)	2,134.7 (2,096.7–2,172.6)	< 0.001
CDAI, mean (95% CI)	0.3 (0.2–0.4)	0.3 (0.2–0.5)	0.2 (0.0–0.4)	0.19
Vitamins A mean (95% CI), mcg	657.8 (638.1–677.5)	665.5 (639.0–692.0)	642.3 (617.1–667.4)	0.21
Vitamins C mean (95% CI), mg	82.3 (79.5–85.2)	85.9 (82.5–89.3)	75.2 (72.3–78.1)	< 0.001
Vitamins E mean (95% CI), mg	9.1 (8.8–9.3)	9.3 (9.0–9.6)	8.6 (8.3–8.9)	< 0.001
Carotenoids mean (95% CI), mcg	10,065.5 (9,664.5–10,466.4)	10,264.0 (9,733.4–10,794.7)	9,662.9 (9,197.9–10,127.8)	0.08
Zinc mean (95% CI), mg	11.7 (11.5–11.9)	11.5 (11.3–11.7)	12.1 (11.8–12.5)	0.004
Selenium mean (95% CI), mcg	113.4 (112.1–114.7)	111.5 (110.0–113.1)	117.2 (114.8–119.7)	< 0.001

NHANES, National Health and Nutrition Examination Survey; NAFLD, non-alcoholic fatty liver disease; CDAI, composite dietary antioxidant index; USFLI, US fatty liver index; PIR, Poverty Income Ratio; BMI, Body Mass Index.

^a^Data are presented as unweighted number (weighted percentage) [95% CI] unless otherwise specified.

^b^NAFLD was defined as USFLI score ≥30.

^c^Race and ethnicity were self-reported.

^d^Included widowed, divorced or separated.

^e^Determined by the survey question, “In any year, have you had at least 12 drinks of any type of alcoholic beverage?”.

^f^Categorized into active/secondhand smoker (>0.011 ng/mL) and non-smoker ( ≤ 0.011 ng/mL).

### Multivariable regression analyses

The results from weighted multivariable regression analyses are presented in Table 2. A negative correlation between CDAI and NAFLD based on USFLI score was revealed in model 1 (crude model) (OR, 0.99; 95% CI, 0.98–1.00), model 2 (OR, 0.97; 95% CI, 0.96–0.99), and model 3 (the fully adjusted model) (OR, 0.95; 95% CI, 0.93–0.98). After transforming CDAI into quartiles, we found that participants with highest quartile CDAI were 34% less likely to have NAFLD than those with the lowest quartile (OR, 0.66; 95% CI, 0.52–0.85), and the trend test was also significant (*P* for trend = 0.002). Furthermore, it is worth noting that the association between CDAI related antioxidant components and NAFLD was also conducted in this study. As shown in Table 2, after adjusted for all variables, vitamin A (OR, 0.85; 95% CI, 0.77–0.95), vitamin C (OR, 0.86; 95% CI, 0.80–0.93), vitamin E (OR, 0.73; 95% CI, 0.62–0.86), and Zinc (OR, 0.79; 95% CI, 0.65–0.97) intake were independently associated with NAFLD.

**Table 2:** Weighted logistic regression analysis on the association between CDAI/antioxidant components and NAFLD.

**Exposures**	**NAFLD defined by USFLI** ^**a**^					
	**Model 1**^b^ **OR (95% CI)**	***P-*** **value**	**Model 2**^c^ **OR (95% CI)**	***P-*** **value**	**Model 3**^d^ **OR (95% CI)**	***P-*** **value**
Continuous CDAI	0.99 (0.98–1.00)	0.19	0.97 (0.96–0.99)	< 0.001	0.95 (0.93–0.98)	< 0.001
**Categories**						
CDAI-Q1	1 [Reference]		1 [Reference]		1 [Reference]	
CDAI-Q2	1.06 (0.91–1.24)	0.44	0.96 (0.82–1.13)	0.64	0.90 (0.74–1.11)	0.33
CDAI-Q3	0.96 (0.82–1.13)	0.63	0.84 (0.71–1.00)	0.05	0.81 (0.63–1.06)	0.13
CDAI-Q4	0.95 (0.81–1.11)	0.52	0.75 (0.63–0.89)	0.002	0.66 (0.52–0.85)	0.002
*P* for trend		0.27		< 0.001		0.002
*ln* (Vitamins A)	0.95 (0.88–1.02)	0.18	0.85 (0.78–0.93)	< 0.001	0.85 (0.77–0.95)	0.003
*ln* (Vitamins C)	0.88 (0.84–0.92)	< 0.001	0.83 (0.79–0.87)	< 0.001	0.86 (0.80–0.93)	< 0.001
*ln* (Vitamins E)	0.87 (0.80–0.95)	0.002	0.79 (0.72–0.87)	< 0.001	0.73 (0.62–0.86)	< 0.001
*ln* (Carotenoids)	0.97 (0.92–1.01)	0.16	0.94 (0.89–0.99)	0.02	0.98 (0.92–1.05)	0.59
*ln* (Zinc)	1.19 (1.07–1.34)	0.002	1.01 (0.89–1.15)	0.90	0.79 (0.65–0.97)	0.03
*ln* (Selenium)	1.34 (1.17–1.51)	< 0.001	1.22 (1.05–1.41)	0.01	0.87 (0.72–1.05)	0.14

NAFLD, non-alcoholic fatty liver disease; USFLI, US fatty liver index; CDAI, composite dietary antioxidant index; OR, odds ratio; PIR, Poverty Income Ratio; BMI, Body Mass Index.

^a^USFLI scores range from 0 to 100, and NAFLD was defined as USFLI score ≥30.

^b^Crude model.

^c^Adjusted for age, sex, race/ethnicity.

^d^Adjusted for age, sex, race/ethnicity, education level, marital status, PIR, BMI, alcohol drinking status, serum cotinine, diabetes, hypertension, metabolic syndrome, and total energy intake.

### Subgroup analyses

**Figure 2:**
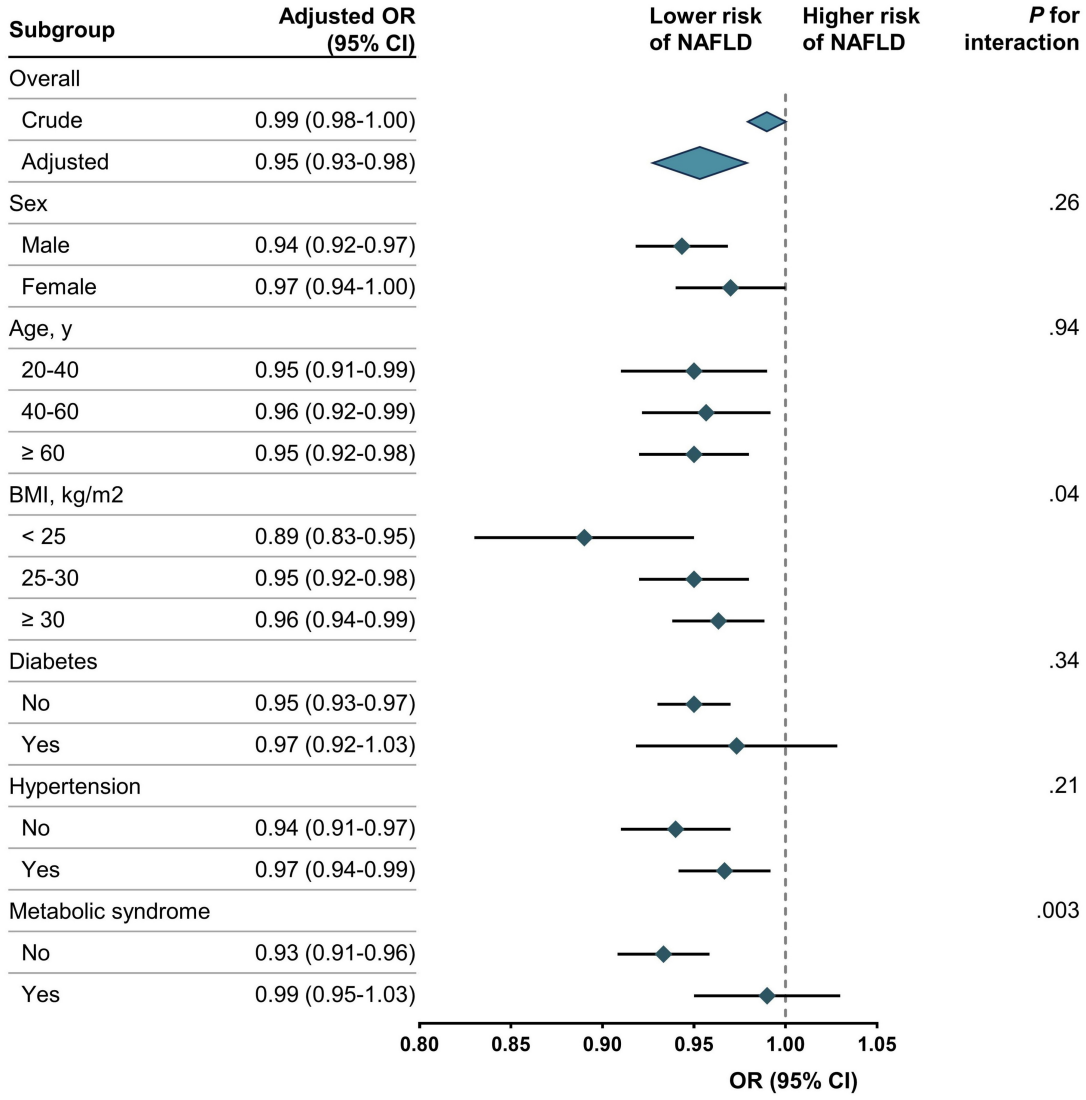
Association between CDAI and NAFLD. NAFLD, non-alcoholic fatty liver disease; CDAI, composite dietary antioxidant index; OR, odds ratio. Each stratification was adjusted for age, sex, race/ethnicity, education level, marital status, PIR, BMI, alcohol drinking status, serum cotinine, diabetes, hypertension, metabolic syndrome, and total energy intake except the stratification factor itself. Rhombus indicates ORs with horizontal lines indicating 95% CIs. Diamonds indicates overall ORs, with outer points of the diamonds indicating 95% CIs.

The results of the subgroup and interaction analyses are shown in Figure 2. CDAI was significantly associated with a lower risk of NAFLD among participants with a BMI < 25 (OR, 0.89; 95% CI, 0.83–0.95) and without metabolic syndrome (OR, 0.93; 95% CI, 0.91–0.96). The interaction tests revealed significant differences in these subgroups (*P* for interaction = 0.04 for BMI and 0.003 for metabolic syndrome). While the negative association between CDAI and NAFLD remained robust across most subgroups stratified by sex, age, BMI, diabetes, and hypertension, the protective effect was strongest among participants with lower BMI and those without metabolic syndrome. In contrast, no significant association was observed among participants with metabolic syndrome (OR, 0.99; 95% CI, 0.95–1.03) or those with a BMI ≥ 30 (OR, 0.96; 95% CI, 0.94–0.99). These findings emphasize that the protective effect of CDAI on NAFLD varies across specific subgroups.

### Sensitivity analyses

The results of sensitivity analyses are summarized in Table 3. After excluding participants treated with methotrexate, acitretin, pioglitazone, liraglutide, semaglutide, atorvastatin, and aspirin (*n* = 8911), CDAI showed negative correlation with NAFLD (OR, 0.95; 95% CI, 0.93–0.98). Following the exclusion of non-Hispanic Black participants (*n* = 7,921), a negative association was observed between CDAI and NAFLD (OR, 0.95; 95% CI, 0.93–0.98), further confirming the robustness of the findings across diverse population groups. Moreover, when NAFLD was defined using the HSI score and adjusted for various covariates including age, sex, race/ethnicity, education level, marital status, PIR, alcohol consumption, serum cotinine, diabetes, hypertension, metabolic syndrome, and total energy intake (*n* = 9,732), the inverse correlation between CDAI and NAFLD persisted (OR, 0.97; 95% CI, 0.95–0.99). This supports the stability of the observed association even when using an alternative definition of NAFLD. Additionally, a sensitivity analysis utilizing unweighted logistic regression (*n* = 9,746) confirmed the stability of the negative association between CDAI and NAFLD (OR, 0.96; 95% CI, 0.94–0.98). Finally, a sensitivity analysis incorporating adjustments for physical activity, measured by MET over a typical week (*n* = 7,677), reaffirmed the robustness of the negative association between CDAI and NAFLD (OR, 0.96; 95% CI, 0.93–0.98).

**Table 3:** Sensitivity analyses.

**Analysis**	**Adjusted OR (95% CI)^a^**	***P*-value**
**Excluding participants taking potential hepatotoxic**		
**medications** ^b^		
Without NAFLD	1 [Reference]	< 0.001
With NAFLD^c^	0.95 (0.93–0.98)	
**Excluding non-Hispanic Black participants**		
Without NAFLD	1 [Reference]	< 0.001
With NAFLD	0.95 (0.93–0.98)	
**NAFLD defined by HSI score** ^d^		
Without NAFLD	1 [Reference]	0.01
With NAFLD	0.97 (0.95–0.99)	
**Unweighted analysis**		
Without NAFLD	1 [Reference]	< 0.001
With NAFLD	0.96 (0.94–0.98)	
**Adjusted for physical activity**		
Without NAFLD	1 [Reference]	0.002
With NAFLD	0.96 (0.93–0.98)	

NAFLD, non-alcoholic fatty liver disease; USFLI, US fatty liver index; HIS, hepatic steatosis index; OR, odds ratio; PIR, Poverty Income Ratio; BMI, Body Mass Index.

^a^Adjusted for age, sex, race/ethnicity, education level, marital status, PIR, BMI, alcohol drinking status, serum cotinine, diabetes, hypertension, metabolic syndrome, and total energy intake.

^b^Included the following medications that may affect liver function: methotrexate, acitretin, pioglitazone, liraglutide, semaglutide, atorvastatin, and asprin.

^c^NAFLD was defined as USFLI score ≥30.

^d^NAFLD was defined as an HSI score ≥36. This model was adjusted for Adjusted for age, sex, race/ethnicity, education level, marital status, PIR, alcohol drinking status, serum cotinine, diabetes, hypertension, metabolic syndrome, and total energy intake.

## Discussion

In this cross-sectional study of nationally representative sample of the US adult population, CDAI was found to be negatively associated with NAFLD in adjusted model. Furthermore, it is noteworthy that the intake of antioxidant components related to CDAI, including vitamin A, vitamin C, vitamin E, and zinc, were also negatively associated with NAFLD. To the best of our knowledge, our study is the first to report a statistically significant negative correlation between CDAI and NAFLD. Moreover, several sensitivity analyses demonstrated the robustness of the relationship between CDAI and NAFLD, affirming the reliability of our study findings. The results of the subgroup analyses further emphasize the complexity of the relationship between CDAI and NAFLD. Specifically, we observed a stronger negative association between CDAI and NAFLD among individuals with a lower BMI (< 25) and those without metabolic syndrome, suggesting that the protective effect of CDAI may be more pronounced in these subgroups. The interaction tests indicated significant differences in the effect of CDAI on NAFLD based on BMI and metabolic syndrome status, underscoring the importance of considering these factors when assessing the impact of dietary antioxidants on liver health. Interestingly, while CDAI maintained a negative association with NAFLD across most subgroups stratified by sex, age, and hypertension, the lack of a significant association among individuals with metabolic syndrome (OR, 0.99; 95% CI, 0.95–1.03) or those with higher BMI (≥30) suggests that the protective effect of CDAI may be attenuated in these groups. These findings highlight the potential for differential effects of CDAI in individuals with distinct metabolic profiles, emphasizing the need for further investigation to elucidate the underlying mechanisms and identify the most responsive populations.

Our findings contribute to an existing body of evidence suggesting associations between the consumption of antioxidant components related to CDAI and lipid metabolism as well as obesity (10, 39, 57). It is noteworthy that, unlike previous studies (16), in our fully adjusted model, we found no significant association between the intake of carotenoids and NAFLD. This finding highlights the potential influence of differing methodologies, populations, or confounding factors in shaping these results. This discrepancy may be attributed to the lack of further categorization of carotenoids in our analysis. It should be noted that NHANES lacks certain carotenoid species significantly associated with NAFLD (58), such as astaxanthin. In our study, the lack of a significant correlation between carotenoids and NAFLD may be also attributed to their potential interaction with other dietary components or demographic factors. When adjusted for age, sex, and race/ethnicity, carotenoids demonstrated a significant negative association with NAFLD. However, after adjusting for all covariates, the negative association remained but was no longer statistically significant. The biological mechanisms underlying the non-significant inverse relationship between carotenoids and NAFLD remain unclear. One potential hypothesis is that carotene may act as an effective antioxidant at lower doses, such as those typically achieved through dietary intake, but loses this efficacy at higher concentrations (59). Notably, although the exact mechanisms are not yet fully understood, a similar pattern has been observed in studies investigating the relationship between carotenoid intake and handgrip strength. In a cohort study by Sahni et al., intake of α-carotene, β-carotene, lycopene, and lutein/zeaxanthin was associated with a reduced loss of grip strength (59). However, in a cross-sectional study by Wu et al., the overall intake of carotenoids was not significantly associated with handgrip strength (35). Additionally, according to the study by Wang et al. (60), there exists a non-linear relationship between serum selenium levels and both ALT activity and the prevalence of NAFLD. Serum selenium levels demonstrate a significant positive correlation when exceeding 130 μg/L, while no significant correlation is observed below 130 μg/L (60). Interestingly, in our study, after stratifying CDAI into quartiles, we found that participants in the highest quartile of CDAI had a 34% lower likelihood of NAFLD compared to those in the lowest quartile (OR, 0.66; 95% CI, 0.52–0.85). Further dose-response studies on antioxidant compounds may be warranted to elucidate the relationship. However, concerning the independent association between intake of vitamin A, vitamin C, vitamin E, and zinc with NAFLD, our results align with several prior studies (61–64). Reactive oxygen species (ROS) and lipid peroxidation directly damage hepatocytes by affecting membranes, proteins, and DNA. Consequently, as antioxidant and anti-inflammatory defenses are depleted, a chronic state of steatohepatitis ensues (65). ROS induces the activation of nuclear factor κB (NF-κB), a key regulator in the synthesis of proinflammatory cytokines, including interleukin-1β (IL-1β), tumor necrosis factor α (TNFα), and interleukin-6 (IL-6) (66–68). The secretion of inflammatory cytokines activates hepatic resident macrophages, such as Kupffer cells and hepatic stellate cells, leading to the infiltration of inflammatory cells and the development of fibrosis. This cascade of events can progress to hepatitis, cirrhosis, liver failure, and potentially liver carcinoma (69). In a prospective cohort study, Luu et al. found that CDAI was inversely associated with levels of IL-1β and TNF-α (70). Traditionally, TNF-α is believed to initiate apoptotic signals in hepatocytes via TNF receptor 1 (TNFR1), which leads to hepatocyte apoptosis, a key feature of NAFLD progression (71). Additionally, recent research by Jin et al. uncovered a positive feedback loop involving macrophage TNF-α-mediated degradation of Myc-interacting zinc-finger protein 1 (Miz1) (72). This degradation results in the inhibition of hepatocyte mitophagy through peroxiredoxin 6 (PRDX6), which exacerbates mitochondrial damage and further amplifies TNF-α production by macrophages. Interestingly, Palladini et al. observed an inverse correlation between serum zinc levels and interleukin-1beta (IL-1β) and tumor necrosis factor alpha (TNF-α) in animal models (73). Furthermore, deficiencies in zinc and vitamin C may exacerbate insulin resistance, thereby increasing the risk of NAFLD (74, 75). These studies may partially elucidate the mechanisms by which antioxidant intake mitigates NAFLD.

While individual nutrients may contribute to the etiology of NAFLD, it is important to acknowledge the potential biological interactions among dietary antioxidants. The notion of an interconnected antioxidant network holds merit, as antioxidants with diverse solubilities are situated in proximal cellular compartments and exhibit the ability to regenerate one another (76). In summary, this study presents a novel approach to exploring factors influencing dietary interventions targeting the reduction of NAFLD incidence. Given the cross-sectional design of this study, we are unable to infer causal relationships, further randomized controlled trials or cohort studies are urgently warranted to validate these findings and offer more precise and effective prevention and treatment strategies for NAFLD Specifically, future research could explore the duration of interventions, appropriate study populations, and potential dietary interventions that could further elucidate the observed associations.

## Limitations

There are several limitations to the present study. First, as a result of the cross-sectional nature of the study, we were unable to construct or confirm any causal inferences. Second, despite the adjustment for potential confounders, residual confounders may still exist which may affect the results. Third, we diagnosed NAFLD in our study using USFLI score, although we conducted sensitivity using HSI score, these non-invasive markers may lack accuracy compared with liver biopsy. Additionally, the use of USFLI >30 as the diagnostic criterion does not allow for the differentiation between NAFL and NASH due to the lack of histological or advanced imaging data. Forth, as the population of this study was American, further studies are needed to determine whether the benefits of dietary antioxidant can be extended to other populations. Finally, recall bias in dietary data may have influenced the accuracy of self-reported food intake, which could affect the findings.

## Conclusion

In conclusion, this cross-sectional study based on six cycles (2005–2016) of the NHANES suggested a negative association between the CDAI and NAFLD in US adult population. This study provides a new approach to explore the factors affecting dietary interventions to reduce the incidence of NAFLD. In the future, well-designed randomized controlled trials are needed to confirm our findings and provide more accurate and effective prevention and treatment options for NAFLD.

## Data Availability

The raw data supporting the conclusions of this article will be made available by the authors, without undue reservation.
